# Assessing the Feasibility and Acceptability of the Objective Structured Clinical Examination (OSCE) in Undergraduate Surgical Training: A Pilot Study

**DOI:** 10.7759/cureus.92992

**Published:** 2025-09-23

**Authors:** Sreejith Kannummal Veetil, Parvez D Haque, Deepak Jain, Mukul Garg, Suchita Rajoria, Binay K Pramanik

**Affiliations:** 1 General Surgery, Christian Medical College and Hospital, Ludhiana, IND

**Keywords:** clinical skills assessment, competency-based medical education (cbme), general surgery education, objective structured clinical examination (osce), undergraduate curriculum

## Abstract

Background: The Objective Structured Clinical Examination (OSCE) is central to competency-based medical education, yet its adoption in resource-limited general surgery settings is underexplored. This pilot study assessed the feasibility, reliability, and acceptability of implementing an OSCE in India’s National Medical Council (NMC)-mandated undergraduate curriculum.

Methods: A prospective cross-sectional study was conducted in a tertiary hospital’s general surgery department over six months. Twenty-eight MBBS students and 15 faculty participated. Three OSCE stations assessed pancreatitis management, Advanced Trauma Life Support (ATLS) primary survey, and history-taking for abdominal pain, using standardized checklists and rating scales. Faculty underwent calibration workshops. Reliability was assessed with Cronbach’s α and Cohen’s κ. Stakeholder feedback was evaluated through structured surveys.

Results: Student performance differed significantly across stations (F = 6.21, p = 0.002); the ATLS station had the highest scores (9.1 ± 0.8). Internal consistency was strong (Cronbach’s α: students 0.84, faculty 0.79), and inter-rater reliability improved post training (κ: 0.42 to 0.78). Female students excelled in communication (p = 0.023). OSCEs caused less stress than traditional exams (p < 0.001), and greater preparation correlated with reduced stress. Student satisfaction reached 92.9%, but faculty reported limited resources (2.9/5). Prior OSCE experience among faculty improved station realism (p = 0.015).

Conclusion: OSCEs in general surgery were feasible, reliable, and well-accepted by students but highlighted the need for better resources, robust faculty training, and institutional support for scalability and sustainability.

## Introduction

The evolution of medical education assessment has been fundamentally shaped by the imperative to move beyond traditional clinical examinations toward more competency-based approaches. The competency-based undergraduate curriculum of the National Medical Commission (NMC) marks a paradigm shift in which various aspects of teaching, including instructional strategies and assessment practices, come together to form the framework of competencies. This creates a foundation that calls for the incorporation of structured assessment methods in order to produce an Indian medical graduate who is sufficiently prepared [1]. This educational transformation builds upon the foundational work established by Harden et al. in 1975, who introduced the concept of the Objective Structured Clinical Examination (OSCE) to address and mitigate the numerous limitations inherent in conventional clinical examination methods. Structured clinical examination allows for more effective regulation of examination variables and complexity, clearer articulation of its objectives, and a broader assessment of the student's knowledge base [2].

The implementation of OSCEs in specialized clinical disciplines has gained significant momentum, as demonstrated by Plöger et al.'s thorough approach to creating OSCEs in obstetrics and gynecology, where they made use of the Delphi technique and Kern's six-step strategy to facilitate organized discussion between highly skilled doctors and guarantee that evaluation procedures are in line with current educational reforms. Building on this implementation framework, the same team's later research demonstrated the viability of interdisciplinary approaches in OSCE development. The planning and implementation of an OSCE in obstetrics and gynecology, which was in line with recent educational reforms, served as a model for other training programs and provided insightful information about practical implementation strategies for various medical specialties [3,4].

The reliability and validity of OSCE assessments have been extensively scrutinized through systematic research approaches. According to a comprehensive systematic analysis of 188 α values from 39 investigations, Brannick et al. reported that the overall Cronbach's α across stations was 0.66. As a result, it was shown that overall OSCE findings often exhibit poor reliability. On the other hand, above-average dependability was linked to more stations and examiners assigned to each station [5]. These psychometric findings have informed subsequent implementation strategies globally, particularly in resource-constrained environments. Gómez-Urquiza et al., in their scoping review, noted that the implementation of OSCE in Latin American healthcare facilities has been repeatedly hampered by the inclusion of inoperative stations, insufficient simulated patient training, and a lack of data supporting the validity of assessment tools [6].

Contemporary developments in OSCE implementation reveal diverse institutional approaches and timing strategies. The national overview of Canadian medical schools by Young et al. revealed significant variation in OSCE practices, with the median number of OSCEs conducted across institutions being four. Formative assessments made up roughly one-third of the total, while summative assessments made up two-thirds. Heterogeneity in curriculum structures and institution-specific resource restrictions are probably the causes of the observed diversity in OSCE implementation [7].

The integration of OSCEs within surgical training programs represents a critical application area where assessment feasibility and acceptability require rigorous evaluation. Given the complex procedural skills, decision-making capabilities, and communication requirements inherent in surgical practice, the implementation of structured clinical examinations in undergraduate surgical training presents both unique opportunities and challenges that warrant systematic investigation through pilot studies examining both feasibility and stakeholder acceptability.

## Materials and methods

Aims and objectives

The primary objective of this study was to introduce OSCE in the Department of General Surgery. The secondary objectives included training faculty members to effectively administer OSCEs and assessing the perceptions of both students and faculty regarding the OSCE as an assessment tool.

Study design and setting

A prospective cross-sectional study was conducted at the general surgery department of Christian Medical College, a tertiary care hospital in Ludhiana, India, over six months (February-July 2025). Stations used standardized checklists and global rating scales. Faculty underwent calibration workshops. Reliability was measured via Cronbach’s α and Cohen’s κ.

Validation of feedback tools

Both the student and faculty feedback surveys were pilot-tested on a separate cohort (n=10) to assess clarity and relevance. Content validity was established through expert review by three medical educators, and internal consistency was confirmed (Cronbach’s α=0.82 for the student tool; α=0.80 for the faculty tool).

Participants

The study population comprised MBBS students enrolled in the general surgery rotation during the study implementation period and surgical faculty actively involved in teaching. Individuals were excluded if they had incomplete attendance during the OSCE training or assessment cycles. Using departmental availability as a guide, a convenience sample of 28 students and 15 faculty members was recruited for the pilot phase.

Development of the OSCE

Three broad OSCEs were developed according to an NMC‑Advanced Course in Medical Education (ACME)‑compliant blueprint that mapped core competencies from the Competency‑Based Surgical Curriculum. Stations 1 and 2 assessed pancreatitis preparation through laboratory and imaging interpretation (OSCE 1, but divided into two stations, one preparatory and the other the actual assessment station). Station 2 involved an Advanced Trauma Life Support (ATLS) primary survey using a high‑fidelity mannequin for trauma resuscitation, and Station 3 focused on abdominal pain history taking with a simulated patient. Each station was allocated three minutes and utilized standardized checklists for assessment. Content validation was performed by three independent surgeon experts, who provided iterative feedback to align scenarios with NMC India guidelines. Faculty calibration workshops were held throughout the six‑month development phase to finalize and standardize station protocols.

Assessment tools and scoring

Stations 1, 2, and 3 were scored using binary checklists (recording each task as “done” or “not done”), while Station 4 employed a global rating scale to evaluate communication skills. Raw checklist and rating scores were converted to a uniform 10‑point scale. Faculty members completed a four‑hour OSCE workshop that covered checklist standardization and protocols for mannequin operation and simulated patient interactions. Inter‑rater reliability was assessed using Cohen’s κ on a 10% sample of randomly duplicated stations.

Feedback collection

After the OSCE cycle, anonymous ACME‑structured questionnaires were administered to both students and faculty (Appendices A, B). The student survey consisted of 10 Likert‑scale items (1 = low to 5 = high) evaluating satisfaction, perceived realism, and stress. The faculty survey mirrored this format with 10 Likert‑scale items assessing satisfaction and resource adequacy, and also included open‑ended questions to capture qualitative insights.

Statistical analysis

Statistical analyses were conducted using IBM SPSS Statistics software, version 26.0 (IBM Corp., Armonk, NY). Descriptive statistics for continuous variables are presented as mean ± standard deviation, and for categorical variables as counts and percentages. Differences in mean performance across the three OSCE stations were evaluated with one-way analysis of variance (ANOVA), followed by Tukey’s post hoc test to identify pairwise contrasts. Group comparisons of continuous outcomes, such as communication performance, stress ratings, and realism scores, were performed using independent two-tailed Student’s t-tests, with degrees of freedom reported in parentheses. Internal consistency reliability of the OSCE checklists was assessed by calculating Cronbach’s α, with values ≥ 0.70 denoting acceptable reliability. Associations between preparation time and student stress were examined using Pearson’s correlation coefficient, and linear regression was used to model predictors of setup delays, reporting the regression coefficient (β), p-value, and coefficient of determination (R²). A significance threshold of p < 0.05 was applied throughout.

## Results

This study examined the implementation of an OSCE cycle involving 28 students and 15 faculty members. As shown in Table 1, all participants completed the assessment (100% retention). Students averaged 23.4 years (±1.2) with 57.1% female representation, while faculty averaged 42.1 years (±8.3) and were predominantly male (87.0%). Prior OSCE experience was limited, with only 17.9% of students and 60.0% of faculty reporting previous exposure.

**Table 1:** Baseline Characteristics of the Participants Descriptive statistics (mean ± SD; n, %) summarizing participant demographics. OSCE: Objective Structured Clinical Examination

Variable	Students (n=28)	Faculty (n=15)
Mean age (years)	23.4 ± 1.2	42.1 ± 8.3
Female, n (%)	16 (57.1%)	2 (13.0%)
Prior OSCE exposure	5 (17.9%)	9 (60.0%)

Table 2 reports mean scores, score ranges, and the results of one-way ANOVA across the three OSCE stations. Station 2 (ATLS primary survey) yielded the highest average score (9.1 ± 0.8), which was significantly greater than Station 1 (pancreatitis management; 8.0 ± 1.2), as indicated by Tukey’s post hoc test (p < 0.01). The ANOVA revealed a significant overall difference among stations (F = 6.21, p = 0.002), suggesting that certain clinical tasks may be more readily mastered or assessed than others. Station 3 (abdominal pain history) produced an intermediate mean score of 8.7 ± 1.0, reflecting robust performance in communication and history-taking skills.

**Table 2:** Station-Wise Performance Metrics One-way ANOVA assessed differences in mean scores across the three stations (F(2,83) = 6.21, p = 0.002). Tukey’s post hoc test identified Station 2 vs. Station 1 as significantly different (p < 0.01). ATLS: Advanced Trauma Life Support

Station	Focus	Mean Score ± SD	Range	F	p
Station 1	Pancreatitis management	8.0 ± 1.2	5.9–9.8	6.21	0.002
Station 2	ATLS primary survey	9.1 ± 0.8*	8.0–10.0		
Station 3	Abdominal pain history	8.7 ± 1.0	7.2–9.9		

**Figure 1:**
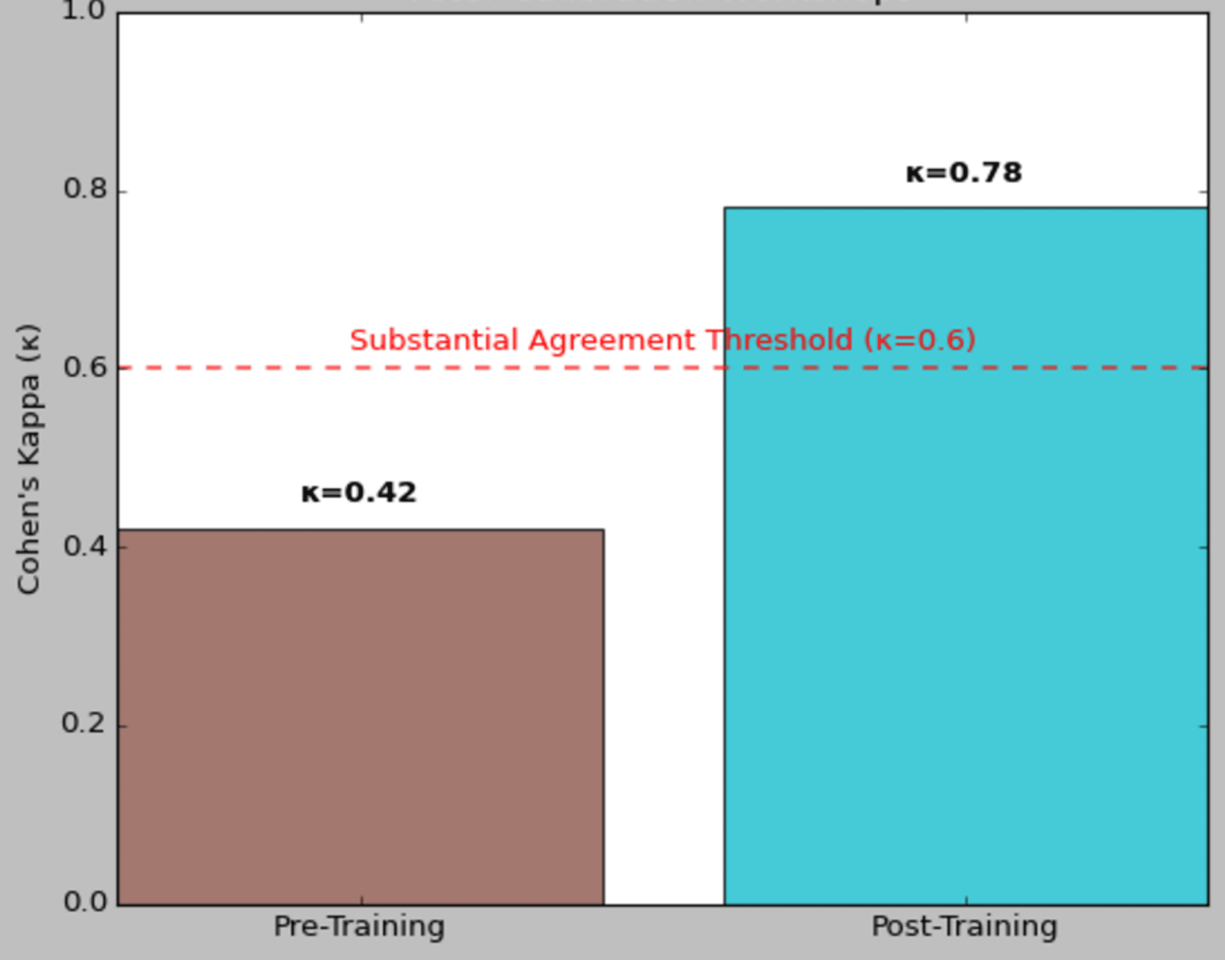
Inter-rater Reliability Improvements Following Assessor Calibration Workshops Internal consistency is reported as Cronbach’s α; inter-rater reliability is Cohen’s κ before and after assessor calibration.

Reliability analysis confirmed that the OSCE checklists demonstrated strong internal consistency and markedly improved inter-rater agreement following faculty calibration. Cronbach’s α indicated high internal consistency for both student evaluations (α = 0.84) and faculty evaluations (α = 0.79) (Table 3). As illustrated in Figure 1, inter-rater reliability κ increased substantially from 0.42 before calibration to 0.78 after calibration workshops, reflecting an upgrade from moderate to substantial agreement among examiners. These findings underscore that structured rater training not only reinforces the coherence of checklist items but also enhances scoring consistency across faculty raters, thereby strengthening the overall reliability of the OSCE assessment.

**Table 3:** Internal Consistency Reliability Metrics Cronbach’s α measured the internal consistency of OSCE checklists. Values ≥ 0.70 indicate acceptable reliability. OSCE: Objective Structured Clinical Examination

Measure	Result
Cronbach’s α (students)	0.84
Cronbach’s α (faculty)	0.79

Table 4 compares group differences on select outcomes using independent two-tailed t-tests. Female students outperformed males on communication performance in Station 3 (t(26) = 2.41; p = 0.023), suggesting a gender-related advantage in patient interviewing skills. Student stress ratings during OSCEs were significantly lower (3.1 ± 0.9) than those for traditional examinations (4.7 ± 0.6; t(27) = 5.27; p < 0.001), indicating that the OSCE format may mitigate test anxiety. Faculty with prior OSCE exposure rated station realism more favorably (4.5 ± 0.3) than OSCE-naïve faculty (3.8 ± 0.6; t(22) = 2.89; p = 0.015), highlighting the impact of familiarity on perceived authenticity.

**Table 4:** Key Comparison Results Independent t-tests compared group means; degrees of freedom reported in parentheses. Two-tailed tests were used for all comparisons. OSCE: Objective Structured Clinical Examination

Comparison	Group 1	Group 2	Statistic
Communication performance (Station 3)	Female: 8.9 ± 0.7	Male: 8.2 ± 1.1	t(26) = 2.41; p = 0.023
Stress ratings: OSCE vs. traditional exams	OSCE: 3.1 ± 0.9	Traditional: 4.7 ± 0.6	t(27) = 5.27; p < 0.001
Station realism (prior OSCE exposure vs. OSCE-naïve faculty)	Prior exposure: 4.5 ± 0.3	OSCE-naïve: 3.8 ± 0.6	t(22) = 2.89; p = 0.015

Table 5 details Likert-scale feedback comparing students and faculty using independent t-tests. Students reported higher overall satisfaction (4.6 ± 0.5) than faculty (4.0 ± 0.4; t(41) = 3.85; p = 0.008) and perceived greater realism in station environments (4.6 ± 0.5 vs. 4.1 ± 0.6; t(41) = 2.67; p = 0.003). Resource adequacy ratings were provided by faculty only (2.9 ± 0.8), indicating moderate satisfaction with logistical support. These findings underscore strong student endorsement of the OSCE experience while identifying areas for enhanced faculty resources.

**Table 5:** Stakeholder Feedback Independent t-tests compared continuous feedback metrics between students and faculty. and score with -Likert Scale: 1 = Low, 5 = High. N/A: not applicable

Metric	Students (n=28)	Faculty (n=15)	Statistic
Overall satisfaction	4.6 ± 0.5	4.0 ± 0.4	t(41) = 3.85; p = 0.008
Realism	4.6 ± 0.5	4.1 ± 0.6	t(41) = 2.67; p = 0.003
Resource adequacy	N/A	2.9 ± 0.8	—

Additional analyses identified a strong negative correlation between preparation time and student stress (Pearson’s r = −0.72; p = 0.002) and a linear regression predicting setup delays (β = 0.82; p = 0.01; R² = 0.68). Faculty with trauma certification rated Station 2 realism higher (4.8 ± 0.2 vs. 4.1 ± 0.5; t(17) = 3.04; p = 0.007).

## Discussion

The OSCE remains a fundamental and widely recognized method for assessing clinical competence in medical education, having been, as described by Khan et al. (2013), initially introduced by Harden in 1975 as a novel alternative to traditional clinical competence assessments. It was specifically designed to improve the validity and reliability of performance evaluations, which had previously relied on long-case and short-case formats [8]. Our findings reveal notable demographic and experiential influences on OSCE outcomes. Female students demonstrated significantly superior performance in communication competencies at Station 3 (abdominal pain history-taking), achieving scores of 8.9 ± 0.7 compared with males’ 8.2 ± 1.1 (t(26) = 2.41; p = 0.023), aligning with prior observations of gender‐related differences in communication skills, though this result should be interpreted cautiously due to potential rater bias and the limited scope of a single station. Faculty experience also emerged as a significant factor influencing station quality, with OSCE‐experienced faculty producing more realistic and credible scenarios (4.5 ± 0.3 vs. 3.8 ± 0.6; t(22) = 2.89; p = 0.015). With 40% of our faculty participants lacking prior OSCE experience, this reinforces the need for dedicated faculty development initiatives aimed at improving both scenario design and assessment fidelity.

Our study reveals significant performance differences across the three stations (F(2,83) = 6.21; p = 0.002), particularly highlighting superior outcomes on Station 2, which assessed the ATLS primary survey (9.1 ± 0.8) compared with Station 1 (pancreatitis management; 8.0 ± 1.2) and Station 3 (abdominal pain history; 8.7 ± 1.0). This disparity underscores the crucial role that station design and task characteristics play in shaping assessment validity and student performance. The algorithmic, protocol-driven nature of the ATLS station likely provided explicit performance benchmarks, boosting student confidence and contributing to consistent scoring. Moreover, this structured format appears to enhance situational awareness, a critical component of clinical competence. As Fischer et al. (2017) noted, whole-task OSCEs enable the evaluation of situational awareness (SA) within the context of clinical reasoning. Notably, the World Health Organization (WHO) has recognized deficient situational awareness as a critical contributor to suboptimal clinical performance [9]. Faculty members with specialized trauma certification rated this station as notably more realistic (4.8 ± 0.2 vs. 4.1 ± 0.5; t(17) = 3.04; p = 0.007), emphasizing how assessor expertise directly influences perceived authenticity and educational impact.

In terms of measurement reliability, our OSCE demonstrated strong internal consistency with Cronbach’s α = 0.84 for students and 0.79 for faculty ratings, both exceeding the 0.70 acceptability threshold and confirming the tool’s robustness. These findings align with Peng et al. (2025), who reported that an OSCE with five to 10 stations, each lasting no more than 10 minutes, has great internal consistency and good reliability. The strongest internal consistency was specifically found in a design that included five to ten stations lasting fewer than 10 minutes each (Cronbach's α = 0.88, 95% CI: 0.86-1.00) [10]. Our three-station design falls within these optimal parameters, and the reliability metrics support Kumaravel et al.’s (2021) findings demonstrating excellent dependability for every summative OSCE circuit (Cronbach's α = 0.67-0.85) [11] in evidence‐based medicine assessments. Nevertheless, significant resource constraints were reported by faculty, with adequacy rated at only 2.9 ± 0.8 on a five‐point scale. These challenges echo Mukurunge et al. (2024), who emphasized that implementing programmatic assessment (PA) into practice requires a lot of resources and time. Thus, a number of structures must be in place for PA implementation to be more faithful [12].

The successful implementation demonstrates what Kumaravel et al. (2021) found. One practical way to evaluate students' evidence-based medicine (EBM) performance and behaviour in a high-stakes assessment environment is to use the OSCEs. The ATLS station’s superior performance supports the efficacy of OSCEs in assessing protocol‐driven clinical competencies and highlights the benefits of spiral curricular models. Kumaravel et al. (2021) also noted that this study is the first to document the usage of several EBM OSCE stations at an undergraduate medical school, where the OSCEs are used to supplement the spiral curriculum of EBM instruction, reinforcing the value of embedding standardized protocols within OSCE frameworks. Furthermore, Suneja et al. (2025) showed that EPA‐based assessments showed that this assessment could produce a moderately reliable score with three cases when using the two rater types and that increasing the OSCE to 12 cases yielded a mean score reliable enough (G = 0.76) for making high-stakes normative decisions regarding remediation and readiness to practice [13].

Stakeholder feedback indicates broad acceptance of OSCEs, with students achieving high satisfaction ratings of 4.6 ± 0.5 compared with faculty satisfaction of 4.0 ± 0.4 (t(41) = 3.85; p = 0.008). Students viewed the format as significantly less anxiety-provoking than traditional exams, with stress levels of 3.1 ± 0.9 versus 4.7 ± 0.6 (t(27) = 5.27; p < 0.001). The strong negative correlation between preparation time and stress (r = -0.72; p = 0.002) suggests that strategic preparatory interventions could mitigate exam‐related anxiety and improve performance.

To address resource constraints, peer‐driven models show promise. Möltner et al. (2020) demonstrated that 94% (67/74) of peer assessors and 90% (276/307) of the peer-assessed group felt that it is important to have peer tutors as assessors, 99% (306/307) were satisfied with their peers as OSCE assessors, and 96% (292/307) considered the peer feedback during the OSCE as helpful. The scale of successful implementation is noteworthy. During the past five years, over 1,500 medical students have passed the formative peer-led OSCE. The two main advantages are that peer tutors as OSCE assessors learn which teaching and learning materials can be adjusted because they have previously taught the students, and peer assessors are more focused (than professional staff) and are not bored during the examination [14].

Near‐peer teaching programs have proven effective, with Rashid et al. (2011) reporting that 96.9% of students say they know what to expect from their final OSCE after attending this program, and 97.9% say the revision course had a positive influence on their learning. One important advantage of cognitive congruence is that near-peer teachers have a better appreciation of the knowledge held by junior peers and can therefore target teaching at an appropriate level due to their close proximity in age and recent similar experiences [15]. Additionally, 73.2% of respondents, junior physicians, provided instruction that was on par with consultant-led instruction, supporting the academic validity of peer‐led approaches. This study reinforces the critical influence of station design, assessor expertise, and resource availability on OSCE effectiveness and reliability. The findings demonstrate that while OSCEs can achieve excellent reliability metrics (Cronbach's α = 0.84 for students, 0.79 for faculty) and high stakeholder satisfaction, successful implementation requires careful attention to optimal design parameters, comprehensive faculty development, and innovative resource management strategies. The evidence strongly supports integrating peer assessment models and near-peer teaching programs to address resource challenges while maintaining educational quality, positioning OSCEs as sustainable and effective tools for clinical competence assessment in medical education.

Limitations

The study’s limitations must be acknowledged. The relatively small sample of 28 students and 15 faculty drawn from a single institution limits generalizability. Limited prior OSCE experience (17.9% of students; 60.0% of faculty) may have introduced novelty bias, potentially affecting stress and perception measures. Evaluating only three stations may not capture the full range of competencies typically assessed in broader OSCE implementations, and unmeasured variables, such as individual study methods or differing faculty instructional experiences, could have influenced results. Reliance on self‐reported feedback also introduces potential bias related to social desirability and recall.

## Conclusions

This pilot study highlights that structured, algorithm-driven OSCE stations, such as the ATLS primary survey, enhance both the reliability of assessments and student confidence. Consistent inter-rater scoring is greatly improved through standardized assessor training, and validated feedback tools show that students perceive OSCEs as fairer and less stressful compared to traditional assessment methods. Faculty members experienced with OSCEs contribute to the development of more realistic and effective stations. For successful OSCE implementation, institutions should adopt evidence-based protocols, involve expert reviews, pilot-test feedback instruments, and provide formal examiner training. While the OSCE format demonstrated reliability and was well accepted, its success is closely tied to the quality of stations, faculty expertise, and resource availability. Performance variability underscores the need for ongoing refinement of stations, and resource limitations highlight the potential benefits of innovative approaches like peer-assisted learning models. This study emphasizes how crucial station design, assessor skill, and resource management are to the OSCE’s overall effectiveness and reliability. It further stresses the importance of faculty development, integrating OSCEs into competency-based curricula, and leveraging peer-led strategies to overcome resource constraints. From both faculty and student perspectives, implementing targeted training programs, promoting collaborative feedback mechanisms, and fostering active learner engagement are key measures to enhance outcomes. These steps are vital to optimizing OSCE delivery, improving learner assessment, and maintaining its central role in clinical education.

## Appendices

Appendix A

**Table 6:** Student Feedback Questionnaire OSCE: Objective Structured Clinical Examination

S. No.	Questions	Strongly Disagree	Disagree	Neutral	Agree	Strongly Agree
1	The instructions at each OSCE station were easy to understand and straightforward.					
2	I felt confident and well-prepared for the OSCE.					
3	The scenarios in each station appeared realistic and relevant.					
4	The simulated patients performed convincingly and effectively (if applicable).					
5	The layout and design of the stations were well-suited to assess the required skills.					
6	The overall environment of the OSCE—including the venue and equipment—met my expectations.					
7	I liked the OSCE better when compared to other methods of assessment					
8	I consider the OSCE a valuable approach for evaluating clinical competencies.					
9	The discussion after the OSCE was helpful					
10	The time allotted for each station was adequate					
11	I felt OSCE accurately assessed my clinical skills					
12	I felt comfortable interacting with the simulated patients					
13	I prefer OSCEs over other assessment methods					

Appendix B 

**Table 7:** Faculty Feedback Questionnaire OSCE: Objective Structured Clinical Examination

S. No.	Questions	Strongly Disagree	Disagree	Neutral	Agree	Strongly Agree
1	I was clear about the process of developing the OSCE stations					
2	I was confident that the OSCE stations were consistently administered among all students					
3	The marking criteria were clear and easy to apply					
5	I felt conducting an OSCE was a tedious process					
6	It is feasible and practical to implement the designed OSCE stations with the available resources at my department (equipment, staff, space)					
7	The checklist used for evaluating students’ performance was clear					
8	The process of implementing the OSCE was easy					
9	The OSCE accurately assessed students’ clinical skills and knowledge					
10	OSCE is an effective method for evaluating the student’s clinical skills					
